# The Zebrafish Embryo as a Model Organism for Testing mRNA-Based Therapeutics

**DOI:** 10.3390/ijms241311224

**Published:** 2023-07-07

**Authors:** Tjessa Bondue, Sante Princiero Berlingerio, Lambertus van den Heuvel, Elena Levtchenko

**Affiliations:** 1 Department of Development and Regeneration, KU Leuven Campus Gasthuisberg, 3000 Leuven, Belgium; tjessa.bondue@kuleuven.be (T.B.); santeprinciero.berlingerio@kuleuven.be (S.P.B.); e.n.levtchenko@amsterdamumc.nl (E.L.); 2 Department of Pediatric Nephrology, Radboud University Medical Center, 6525 GA Nijmegen, The Netherlands; 3 Department of Pediatric Nephrology, Emma Children’s Hospital, Amsterdam UMC, 1105 AZ Amsterdam, The Netherlands

**Keywords:** zebrafish, mRNA therapy, micro-injection

## Abstract

mRNA-based therapeutics have revolutionized the world of molecular therapy and have proven their potential in the vaccination campaigns for SARS-CoV2 and clinical trials for hereditary disorders. Preclinical studies have mainly focused on in vitro and rodent studies. However, research in rodents is costly and labour intensive, and requires ethical approval for all interventions. Zebrafish embryonic disease models are not always classified as laboratory animals and have been shown to be extremely valuable for high-throughput drug testing. Zebrafish larvae are characterized by their small size, optical transparency and high number of embryos, and are therefore also suited for the study of mRNA-based therapeutics. First, the one-cell stage injection of naked mRNA can be used to assess the effectivity of gene addition in vivo. Second, the intravascular injection in older larvae can be used to assess tissue targeting efficiency of (packaged) mRNA. In this review, we describe how zebrafish can be used as a steppingstone prior to testing mRNA in rodent models. We define the procedures that can be employed for both the one-cell stage and later-stage injections, as well as the appropriate procedures for post-injection follow-up.

## 1. Introduction

A wide array of diseases are caused by protein absence or dysfunction due to a genetic defect. Messenger RNA (mRNA)-based protein replacement therapies have recently gained attention in the treatment of genetic diseases as they can result in the prompt re-expression of a functional protein and thereby restore gene function. Therefore, mRNA-based protein replacement can be curative for a multitude of genetic disorders that do not currently have a (curative) treatment available. The mRNA-based approaches have some advantages in comparison to DNA-based gene replacement using viral vectors (Table 1). First, mRNA administration results in rapid protein expression upon entrance in the cytosol without the need for nuclear translocation. As there is no risk of insertional mutagenesis due to genomic integration, mRNA-based therapeutics are considered to be safe, while the transient expression allows for dose flexibility. Finally, similar to DNA-based approaches, mRNA-based therapeutics are not mutation specific with one product being suitable for the entire mutational spectrum of the disease. The use of mRNA in the clinics was historically limited due to its instability and immunogenicity. This limitation has been tackled in recent years and the mRNA therapies that are currently being tested in clinical trials use a modified mRNA structure that results in improved stability and reduced immunogenicity [1]. Additionally, non-viral delivery vehicles are often employed to protect the mRNA from degradation and enhance (organ-specific) cellular uptake. These delivery vehicles have several advantages as opposed to viral vectors that are typically used for DNA-based gene therapy, mostly related to their standardized and cost-effective manufacturing process and decreased immunogenic potential (Table 1) [1,2,3].

In this review, we describe the use of zebrafish models in the study of mRNA-based protein replacement strategies and compare it with the use of rodent models. We discuss in detail the techniques that are used to test mRNA-based therapies in zebrafish, illustrated by several examples from the literature, and propose the steps required to extrapolate data obtained in the zebrafish models to the more complex animals.

## 2. Why a Zebrafish Model?

Zebrafish (Danio Rerio) are commonly used in biomedical research and have been extensively employed as a step up from in vitro preclinical studies and a precursor to more advanced studies in rodent models, with each of these models having their own pros and cons (Table 2). Zebrafish are particularly useful for developmental studies due to their external development and the high number of embryos produced in a single round.

Furthermore, the increasing toolbox for genetic modification in the zebrafish, which has approximately 70% of homology with the human genome, has resulted in the generation of a multitude of zebrafish disease models to study pathogenic mechanisms and for high-throughput drug testing [4,5]. According to the European Commission Directive (2010/63/EU) and the commission implementing decision (EU) 2020/569, experiments with zebrafish larvae before the independent feeding stage (<5 days post-fertilization) are not regulated as experiments on laboratory animals. Therefore, work with 0–5 days post-fertilization larvae can be considered as an alternative to animal studies. However, regulations differ between countries and these issues should be consulted before starting the study. Also, measurements in the fish water can be used as a proxy for evaluating kidney function, similar to urine analysis in humans [6]. In contrast with rodent models, zebrafish allows for more flexibility to test therapeutic strategies, thereby reducing costs and time [7]. Furthermore, due to its rapid development many disease phenotypes will already be distinguishable within this short timeframe and can thus be used to study therapeutic efficiency in a high-throughput manner [8].

**Figure 1:**
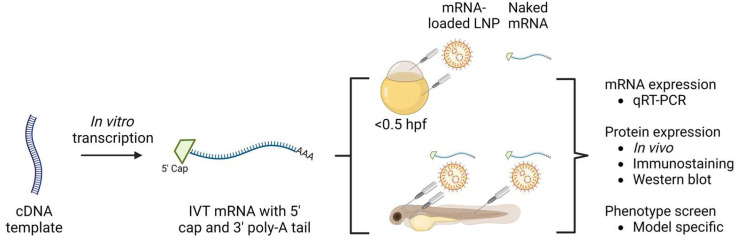
Evaluation of mRNA-based therapies in the zebrafish model. Synthetic mRNA can be produced with desired modifications by in vitro mRNA transcription (IVT mRNA) from a cDNA template with the sequence of interest. Modified IVT mRNA is equipped with a 5′ cap and 3′ poly-A tail and can be injected in the one-cell stage as naked mRNA or packaged in a delivery vehicle. mRNA can also be injected into older zebrafish embryos. After injection, mRNA can be detected and protein expression confirmed. Phenotypic screens can be carried out post-injection to assess effectiveness. hpf = hours post-fertilization. This figure was created with BioRender.com.

While rodents are the most widely used animal species to study mRNA-based therapeutics, genetic material can already be injected into the one-cell stage zebrafish embryo. This is more cost- and time-effective as compared with the rodent models and eliminates the need for a delivery vehicle at this stage of the study. Furthermore, older zebrafish embryos (>24 h post-fertilization (hpf)) have a functioning circulation system and can be injected locally or systemically (Figure 1) [9]. To establish targeted mRNA therapies in the rodent model, extensive research needs to be executed to develop the ideal delivery vehicle. In this regard, zebrafish can serve as an intermediate step and narrow down the options for rodent testing [10]. Here, we provide an overview of how to approach the testing of mRNA-based therapeutics in zebrafish, supported by examples from the literature.

## 3. In Vitro Transcribed (IVT) mRNA

**Figure 2:**
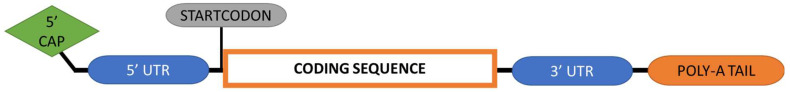
The structure of a functional mRNA molecule. A functional mRNA model is capped on the 5′ side to protect from exonuclease attack. The coding sequence is flanked by the 5′ and 3′ untranslated regions (UTRs), which influence translation efficiency and stability. Finally, a long stretch of adenine residues (poly-A tail) at the 3′-end provides further stabilization and is a main determinant of mRNA half-life.

mRNAs can be obtained from manufacturing companies or be synthesized in house by in vitro transcription (IVT) from a DNA template (Figure 1). In the DNA template, the following components are coded: (1) the sequence of interest, (2) the 5′ and 3′ untranslated regions (UTRs) and (3) a promotor for the RNA polymerase system (bacteriophage T7, T3 and SP6 promotors are the most widely applied). Finally, a functional mRNA molecule is further equipped with a 5′ cap structure and a 3′ poly-A tail (Figure 2). These components can be added during the transcription by adding capping analogues and encoding a poly-T stretch in the DNA template, or afterwards by capping enzymes and poly-A polymerases [1,2]. Importantly, the above-mentioned building blocks of IVT mRNA can be modified in order to improve stability and translation efficiency, and decrease immunogenicity [1].

### mRNA Modifications Influence Expression Levels in Zebrafish

In zebrafish, IVT mRNA has been used for ectopic expression or overexpression experiments to study gene function after injection at the one-cell stage and modified IVT mRNA has been applied to manipulate the expression levels [10]. It is worth noting that zebrafish can tolerate mRNA sequences from other species, which can increase the cost-effectiveness of a project [11,12,13].

Several modifications in the coding sequence, such as the use of pseudo-uridine instead of uridine, have shown promise in reducing immunogenic potential by preventing recognition of the mRNA by the mammalian innate immune system, which happens via toll-like receptor 7 (TLR7) and retinoic-acid-inducible gene I (RIG-I) [14]. The expression of TLR7 and RIG-I orthologues has been demonstrated in zebrafish, but studies using human mRNA in the zebrafish embryo have not provoked any toxicity or any signs of immune response after injection, as will be illustrated below [11,13,15]. Next, the use of artificial UTRs has been shown to increase protein expression when compared to the endogenous UTRs from the zebrafish genome. Fink et al. studied the effect of different UTRs by generating luciferase constructs, with either artificial UTRs or endogenous UTRs of the development gene *pax2*. They observed a decrease in expression of the construct using the *pax2* UTRs, suggesting a destabilizing effect. This observation might be extrapolated to other developmental genes that, like *pax 2*, play a role in early development and are therefore only active in the embryo for a short period of time [10]. In addition, a functional mRNA requires a GTP-based 5′cap to protect from exonuclease activity. In mammals, several types of capping analogues have been described and IVT mRNA with anti-reverse capping analogues (preventing reverse integration of the cap upon addition to the IVT mRNA reaction) has been used in zebrafish [16]. Finally, shortening of the poly-A tail with every translation round is a main determinant for the mRNA stability in mammalian systems [17,18]. In the same study, Fink et al. also demonstrated that using a SV40 poly-A tail sequence increased expression of mRNA in the early zebrafish embryos but only slightly influenced the stability. Similarly, the inclusion of a 148A-tail showed the highest expression of *GFP* in a study by Linares-Fernández et al. following one-cell stage injection [10,16].

## 4. Evaluating mRNA-Based Therapies in the Zebrafish

### 4.1. Selection of an Appropriate Zebrafish Model

Comparison of the zebrafish and human reference genome shows that approximately 70% of human genes have a zebrafish orthologue. In the past, a wide range of disease models were generated by means of random mutagenesis, using N-ethyl N-nitrosurea (ENU) and subsequent phenotypic evaluation. Recent advances in targeted nuclease technologies (zinc finger nucleases—ZFNs, transcription activator-like effector nucleases—TALENs and sgRNA guided CRISPR-Cas9) have allowed for a more rapid and specific introduction of damaging mutations in a specific gene of interest [19].

When designing a zebrafish model, the zebrafish orthologue of a gene of interest (disease causing gene) needs to be identified. Importantly, the zebrafish has undergone a whole genome duplication during evolution, resulting in at least two zebrafish orthologues for many human genes [20]. However, some of the duplicated genes are not functional. Therefore, only the functional orthologues need to be edited. Next, the generated disease models should be phenotypically characterized and a suitable readout determined.

The pathogenesis of the disease itself will determine which mRNA can be used. At this moment, only a few zebrafish models of monogenic diseases have been used to study mRNA-based therapies (Table 3). While diseases caused by mutations in more than one gene could in theory be treated by combining different mRNAs in one injection/vehicle, this has not been explored yet in the zebrafish. Also, the therapeutic effect can be obtained by using genes that play a role in the pathogenesis rather than the causative genes (Table 3) [12,21].

### 4.2. The Injection of mRNA in the One-Cell Stage Embryo

Once a reliable disease model is available, mRNA can be tested to restore or attenuate the phenotype. In order to test the therapeutic effects of synthetic mRNA in zebrafish, micro-injection at the one-cell stage is the most widely applied method. At this stage, there is only one blastomere, which takes up the mRNA via cytoplasmic flow and rapidly divides, resulting in ubiquitous expression in all cells of the embryo [22].

#### 4.2.1. Experimental Setup: One-Cell Stage Injection of mRNA

For one-cell stage injection of mRNA, the mRNA is dissolved in RNAse free water and kept on ice during the procedure. Furthermore, it is recommended to add 0.05% of phenol red, a red-coloured dye, to make needle calibration and injection more practical. Embryos are collected after fertilization and need to be injected within 30–45 min post-fertilization. If injection is performed later, the mRNA will no longer be divided over all cells and result in mosaicism [4,22]. Injection needles can be bought readymade but can also be made by researchers themselves from glass capillaries with a micropipette puller. Before injection, it is of crucial importance to calibrate the injection volume. This can be performed with the phenol red in a drop of mineral oil on a micrometer (0.1 mm = 500 pL). Once needles are calibrated, eggs are oriented on a solid support with the yolk facing the end of the needle. After loading the needle with mRNA solution, the chorion and yolk of each egg is penetrated and the mRNA injected by a short pressure wave. After injection, the needle is carefully removed and the zebrafish left to further develop at 28 °C in E3 medium [22,23].

#### 4.2.2. Current Applications

Several studies have employed one-cell stage injection to show phenotype restoration by means of mRNA-based protein replacement/addition. One of the first reports in 2014 described a zebrafish model of Duchenne muscular dystrophy (DMD), a muscle-degenerative disease caused by mutations in the dystrophin gene, which is also expressed in the zebrafish skeletal muscle. The protective effect of heme-oxygenase has been studied before in mouse DMD models [24] and the one-cell stage injection of *hmox1* mRNA confirmed a significant restoration of the DMD phenotype in the zebrafish model [21]. More recently, the one-cell stage injection has also been used to study phenotype restoration in a model of Wolfram syndrome, a rare neurodegenerative disease caused by mutations in the Wolframin (*WFS1*) gene. Wolframin protects NCS1, a Ca^2+^-sensor, from degradation and regulates the ER-mitochondria Ca^2+^ transfer. Crouzier et al. established a zebrafish model of Wolfram syndrome (*wfs1ab^KO^*) and demonstrated that the one-cell stage injection of murine *Ncs1* mRNA can restore mitochondrial function and hyperlocomotion of the zebrafish [12]. Additionally, a zebrafish model of classic galactosemia (CG) (*galt^KO^)*, a hereditary disease of galactose metabolism caused by deficiency in galactose-1-phosphate:uridylyltransferase (GALT) activity, has been treated with human *GALT* mRNA by one-cell stage injection, resulting in enzyme activity and a decrease in the accumulated metabolites [11]. Finally, our group treated a zebrafish model for cystinosis (*ctns^−/−^*), an autosomal recessive lysosomal storage disorder caused by a defective or absent lysosomal cystine transporter (cystinosin), and resulting in cystine accumulation, with human *CTNS* mRNA at the one-cell stage and showed restoration of the kidney phenotype (proteinuria and proximal tubular reabsorption) along with reduction of whole body cystine levels [13].

These pioneering studies illustrate that naked mRNA injection is the most straight-forward strategy applied in zebrafish. While the use of delivery vehicles is crucial in more complex organisms like rodents, they are not often used in zebrafish for one-cell stage injection. Moreover, packaged mRNA has been shown to be less efficient at obtaining high levels of protein expression in comparison with the injection of naked mRNA at the one-cell stage [25]. Therefore, we recommend using naked mRNA as the first step for evaluating the effectiveness of mRNA-based therapies.

### 4.3. The Injection of mRNA in the Later-Stage Embryo

As discussed, one of the main challenges in mRNA-based medicine is the need for an appropriate delivery vehicle. Few studies have used delivery vehicles loaded with mRNA in zebrafish as rodent models are the most commonly used for this purpose. The standard delivery vehicles (such as lipid nanoparticles) are mostly taken up by the liver and extra modifications are needed if other organs need to be reached. Such modifications and targeting strategies are currently the most important challenge in the development of mRNA-based protein replacement therapies [1]. In recent years, efforts have been made to develop mRNA delivery vehicles to protect the mRNA from degradation and ensure efficient delivery that can also be tested in zebrafish [1].

#### 4.3.1. The Importance of Choosing the Right Injection Strategy

**Figure 3:**
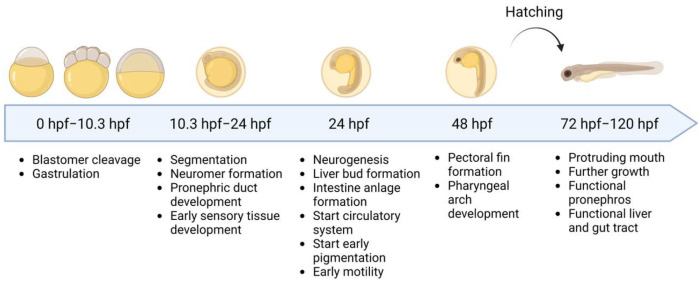
Early development of the zebrafish embryo. After fertilization, the blastomere undergoes cleavage and gastrulation. Between 10.33 hpf and 24 hpf, the somites are formed (segmentation) and, at the 13-somite stage (24 h), the pronephros begins to form. The circulatory system can be seen by the presence of a heartbeat and organogenesis continues with the formation of the neuromeres, otoliths, liver anlage and intestine anlage. At 48 h, pectoral fins are formed and the embryo is touch reactive. Hatching occurs between 48 hpf and 72 hpf, and the early organogenesis is finalized. The free-swimming embryo has a protruding mouth with a functional pronephros and intestinal tract. Intravenous delivery can be performed from 24 h–48 h onwards. This figure was created with BioRender.com.

Protocols for injecting in later-stage embryos can vary significantly based on the research question, which will partly determine the most suitable route of injection. Several locations have been utilized to introduce genetic material into the zebrafish embryo, such as hindbrain ventricle, caudal vein and trunk injection [11,25]. As most studies aim to bring mRNA therapy to the clinical setting, some injection routes that are used in zebrafish are less desirable for human applications. The transient nature of mRNA expression implies the need for repeated dosing and, therefore, some local injection strategies (for example into the brain [25]) cannot be extrapolated to a clinical setting. Therefore, most studies aim to establish systemic injection of the mRNA. In the zebrafish, blood circulation begins by 24 hpf (Figure 3) [26] and intravenous injection of (packaged) mRNA is possible from 24–48 h post-fertilization onwards [27,28].

Lipid based nanoparticles (LNPs) are one of the most attractive vehicles for mRNA delivery and are mainly taken up via the LDL-receptor in the liver [29]. Notably, LDL-receptor expression in zebrafish is low at 24 hpf but increases over time. Hence, it is indeed preferential to use naked mRNA in early embryos (<24 hpf) and use a lipid-based delivery vehicle in older embryos [11]. Importantly, current research is mainly focused on finding delivery vehicles to target other organs and circumvent sequestration in the liver. For this, LNP composition is refined and targeting moieties are used. Often, multiple formulations are manufactured and need to be tested to select the best targeting strategy. The zebrafish embryo can serve as a potential model organism to evaluate the targeting capabilities of different vehicle formulations. By testing different components in zebrafish first, the number of vehicle formulations to be tested in the rodent models can be reduced.

#### 4.3.2. Experimental Setup: Intravenous Injection into the Duct of Cuvier

**Figure 4:**
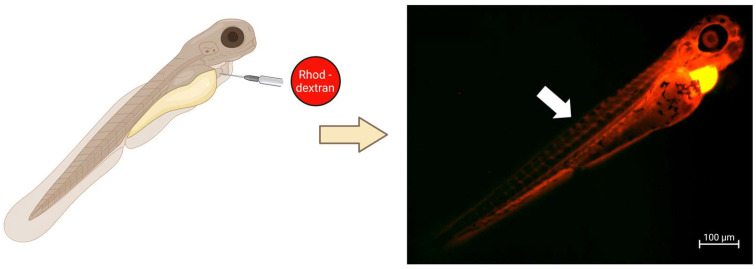
Duct of Cuvier injection results in circulation of the injected solution. The 72 h-old embryos were injected in the duct of Cuvier with a fluorescence-labelled molecule (rhodamine B isothiocyanate–dextran) and evaluated with fluorescence microscopy. Successful injection can be validated within a few minutes as the presence of labelled compound in the circulatory system (arrow). This figure was created with BioRender.com.

For intravenous injection, (packaged) mRNA formulations can be used in combination with 0.05% phenol red and needle calibration has to be performed. Importantly, at >24 hpf the zebrafish embryos have increased motility, which can impair the efficiency of injection. Therefore, duct of Cuvier injection is performed after anaesthetizing the embryos with tricaine methane sulfonate (MS222-200 μg/mL) and orienting them on an agarose grid, with the heart pointing to the end of the needle. Injection in the duct of Cuvier will result in systemic circulation, as this is a wide circulation channel connecting the heart and trunk vasculature. We recommend practicing this injection technique by using fluorescent molecules that allow the direct assessment of the systemic circulation of the injected product. Figure 4 illustrates the expected distribution pattern after injection in the duct of Cuvier using a rhodamine B isothiocyanate–dextran at 72 hpf. Once mastered, injection is performed by penetrating the heart sac with the needle. Ejection of the mRNA solution can be recognized by using phenol red, which will be visible around the injection site, and by checking for volume expansion after the injection pulse [30].

#### 4.3.3. Current Applications

As mentioned above, injection of mRNA in older embryos to restore a disease phenotype has not been widely used. Nevertheless, systemic injection of *GFP* mRNA via the caudal vein has been shown to result in GFP expression in the tissues close to the injection site, with other injection routes showing altered patterns of expression. Also, Pattipeiluhu et al. showed that the injection of anionic LNPs in the duct of Cuvier delivered *GFP* mRNA and fluorescently labelled mRNA to sinusoidal endothelial cells and macrophages [29]. The same study also showed the biodistribution of the FDA-approved DSPC-LNP in the zebrafish, resulting in non-specific mRNA delivery in a wide array of cell types in young larvae (<48 hpf) and the ApoE-mediated uptake of liposomes in older embryos (>72 hpf) [29]. The systemic injection in the duct of Cuvier was used in one study to evaluate the effect of LNP-packaged *GALT* mRNA in a model of classic galactosemia, which resulted in increased enzyme activity and reduced accumulation of metabolites as described in Table 3 [11]. However, organ localization of the injected mRNA was not studied. Furthermore, classic galactosemia is a disease that primarily affects the liver, which is naturally equipped to take up LNPs. Therefore, we can presume that the LNP used in this study was not modified to target other organs.

### 4.4. Assessment of Protein Expression and Effectivity after Injection

#### 4.4.1. Validation of mRNA Injection with qRT-PCR

mRNA levels can be quantified shortly after injection to assess the successfulness of the injection. The mRNA can be isolated from the embryos and cDNA can be synthesized, followed by a quantitative reverse transcription polymerase chain reaction (qRT-PCR) with specific primers. However, this approach is not widely employed, as investigators are mainly interested in protein expression, which occurs rapidly after uptake of mRNA by the cells. Also, while qRT-PCR can be used to assess mRNA half-life, this is not always relevant and resources are mostly focused on mapping out protein half-life. Nevertheless, our study of *CTNS* mRNA in cystinotic zebrafish (*ctns^−/−^*) has detected mRNA for at least 120 h post-injection [13].

#### 4.4.2. Assessing Protein Expression after mRNA Injection

While phenol red and/or fluorescent markers can give a good first impression about the successfulness of the injection, confirming functional protein expression is the most important experimental step. When using the one-cell stage injection method, the protein is expected to be ubiquitously expressed. This can be studied by classic methods like Western blotting and immunostaining [11,13]. When suitable antibodies are available, it is recommended to start by assessing the expression in the whole embryo by Western blot, providing amplification of the signal by combining multiple embryos in a single lysate. After protein expression has been confirmed, localization can be further assessed by immunostaining on embryo sections. However, the latter has not been performed in the zebrafish larvae yet. While in rodent studies, the subcellular localization of the protein can be determined after dissection of the animal; this is less feasible in zebrafish because of the small size of the embryos. However, dissection of larger individual organs (such as eye, intestine, heart and brain tissue) has been performed in zebrafish larvae between 72 hpf and 120 hpf [31,32,33,34]. Immunohistochemistry and immunostaining are mainly recommended when using packaged mRNA in later-stage embryos to assess the targeting potential of the delivery vehicles, as the one-cell stage injection is presumed to result in ubiquitous expression. However, it must be noted that current studies have only assessed tissue distribution with vehicles loaded with fluorescent proteins (such as GFP [25]), while whole embryo lysate based Western blots were used to confirm overall protein expression when using non-fluorescent proteins (such as GALT [11]). Based on this, we cannot state with certainty that immunostaining for non-endogenously fluorescently tagged proteins after injection of mRNA-loaded LNPs is the most effective way to show protein tissue distribution and recommend using a strong fluorescent protein to assess the targeting efficiency of delivery vehicles. Importantly, protein expression is not only dependent on delivery but also on translation capacity that can differ from tissue to tissue. Therefore, the delivery vehicle itself or the encapsulated mRNA could also be fluorescently labelled in order to assess only the biodistribution, regardless of the translation of the protein itself [29,35].

#### 4.4.3. Evaluation of Reporter Gene Expression by Live In Vivo Microscopy

**Figure 5:**
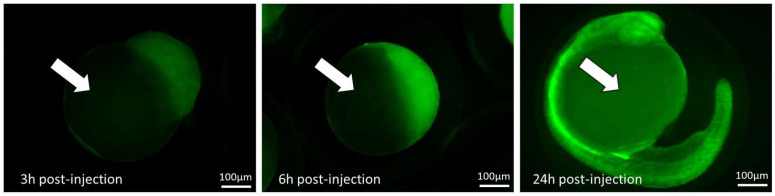
The mRNA’s coding for a fluorescent protein can be used to follow up protein expression during development. Synthetic mRNA that codes for a GFP was injected at the one-cell stage and fluorescence followed up during the first 24 h post-injection. The GFP protein can be seen in the blastomeres within 3 h post-injection and in the embryo body at 24 h post-injection. As no translation happens in the yolk (arrow), there is no protein observed there. As expected with one-cell stage injection, the protein expression is ubiquitous. Scalebar = 100 µm.

As discussed, one of the major advantages of the zebrafish embryo is the optical transparency, which can be exploited to follow protein expression in the developing embryo by encoding a reporter gene tag in the IVT mRNA. In practice, green-fluorescent protein (GFP) tags are used most often, unless the model already expresses a GFP-labelled product and other fluorescent tags (such as red-fluorescent protein (RFP) [13]) need to be explored. The use of mRNA encoding a fluorescent protein (tag) will result in a detectable fluorescence reflecting the expression of the protein, which can be followed in the live embryo by fluorescence microscopy (Figure 5). Notably, when using late-stage injection, the expression is not expected to be ubiquitous and organ localization can be performed, as shown with hindbrain injection of *GFP* mRNA [25].

#### 4.4.4. Evaluation of Toxicity in Zebrafish Embryos

Zebrafish embryos are often used as an early in vivo model for toxicity screening of candidate drugs. One of the most robust methods for assessing toxicity after one-cell stage injection of mRNA is to evaluate embryo survival. Additionally, the study of teratogenicity can be carried out to quantify developmental toxicity by observation of the embryo morphology. Typical indicators of developmental toxicity include delayed growth, restricted movement, curved spine, and yolk sac or pericardial oedema. Additionally, embryo toxicity can be studied by late-stage evaluation of heart rate, blood circulation and motility, with the latter being tightly correlated with the general morphology of the fish (for example, a severely curved spine will lead to an abnormal circular movement of the zebrafish larvae) [36]. Strikingly, for the studies mentioned in Table 3, no overt signs of toxicity have been observed for embryo mortality rate and/or morphology (irregular head–trunk angle and pericardial oedema) [11,13]. Additionally, while hatching rate is often used to assess the developmental toxicity of a variety of drug compounds [36], it has not been studied for mRNA injections, possibly related to the injection itself influencing the hatching rate.

#### 4.4.5. Phenotype Evaluation of mRNA-Based Therapies in Zebrafish Embryos

As illustrated, only a few studies have applied mRNA-based protein replacement in the zebrafish model. So far, mRNA injections are mainly used to identify protein function or to generate disease models. However, the above-mentioned studies show the potential of these approaches to establish effectiveness [37]. Depending on the disease that is studied, different phenotypes can be evaluated after injection of mRNA and the phenotypes are therefore model specific. Notably, the developmental stage at which the phenotype can be studied also needs to be considered. As most organ systems develop after 24 hpf, earlier assessment of particular phenotypes will mostly not be possible. In practice, most phenotypes have been studied in 4–5 day (96 hpf–120 hpf) old fish. For example, the classic galactosemia model (*galt* knockout) was used to assess the effectiveness of the *GALT* mRNA injection by quantification of GALT enzyme activity and metabolomics at 5 days post-injection [11]. Also, the *wfs1ab* knockout fish model of the Wolfram syndrome phenotype was studied for locomotion (motor response to light–dark sequence) and mitochondrial function (oxygen consumption rate with the Agilent Seahorse XF Cell Mito Stress test) at 5 days post-injection of murine *Ncs1* mRNA [12,21]. Finally, proteinuria and proximal tubular reabsorption after one-cell stage injection of *CTNS-mCherry* mRNA into the *ctns^−/−^* zebrafish model was studied by our group at 5 and 4 days post-injection, respectively [13]. When early developmental stage injection is performed, it is less desirable to assess a phenotype that is only present in the adult fish. While exact timeframes of effectiveness in zebrafish have not been explored, the transient nature of mRNA-based therapeutics suggests that, after embryonic injection, the effect will not always be present in adult fish. Nevertheless, when the DMD *sapje* zebrafish was used to evaluate the effect of *hmox1* mRNA overexpression on anti-myosin heavy chain expression, an amelioration was observed at 20 days post-injection. However, in this case it must be noted that zebrafish muscle development starts early and that all 30 myotomes are already present by 24 hpf [38].

## 5. From Zebrafish to Higher Mammals

Although zebrafish are phylogenetically more distant from humans than rodent models, they exhibit a high degree of physiological and pharmacological similarities that can be used for the study of therapeutic strategies. Most molecules that are active in zebrafish have a similar effect in mammal and human systems, and many small molecules have shown disease rescuing activity in the zebrafish and subsequently successfully been extrapolated to clinics [37]. However, the reliability of the effects of the mRNA-based therapy and extrapolation to more complex models depends on the representativeness of the model and the physiological pathways that are targeted [39].

As mRNA-based therapeutics have only recently risen to prominence, no such therapeutics have been tested in the zebrafish model and moved onto rodent models. So far, only one study with (packaged) mRNA-based protein re-expression has been performed in both zebrafish and mammal models independently. Strikingly, both these animal models showed similar outcomes. The classic galactosemia mouse model showed that systemic administration of packaged *GALT* mRNA resulted in the expression of the enzyme in the liver. Similarly, LNP-packaged *GALT* mRNA was injected in a *galt^KO^* zebrafish model as well and resulted in functional enzyme expression [11,40]. This observation further underlines the potential advantage of using zebrafish models as a partial replacement for the rodent studies. The zebrafish has been used in parallel to rodent models to evaluate the LNP distribution pattern, with striking similarities. Indeed, Pattipeiluhu et al. recently highlighted the position of the zebrafish as a model organism to study the uptake of the zwitterionic lipid containing LNPs versus anionic LNPs in the liver reticuloendothelial system, showing the stabilin dependent preferential uptake of anionic LNPs in the sinusoidal epithelial cells of both the zebrafish embryo (96 hpf) and a mouse model [29].

## 6. The Limitations of Using Zebrafish

There are some challenges that need to be taken into account when using the zebrafish for mRNA-based therapy studies. First of all, some inheritance disadvantages exist with using zebrafish as compared to higher animals. As the last common ancestor between humans and zebrafish lived about 445 million years ago, the increased evolutionary distance results in extra effort being needed to develop functional assays to study specific human-like phenotypes. Also, their anatomical differences can make them unsuitable for studying some physiological parameters. As an example, the study of degenerative diseases can be hampered in zebrafish, as they have a regenerative capability that needs to be taken into account when studying the effectivity of mRNA re-expression. As mentioned above, the whole genome duplication that occurred in evolution results in several human genes having two zebrafish counterparts. Therefore, when developing models for testing mRNA-based therapeutics, one needs to be sure that all active paralogues are addressed, as these might still result in residual gene function that will affect the dose of mRNA needed for a full phenotype rescue. However, more studies are required to provide additional experimental data for these issues. Finally, compound delivery by diffusion can be hampered by the presence of the chorion. However, as mRNA is directly injected into the embryo, this point is of little relevance for mRNA-based therapeutics [41].

As mentioned above, one-cell stage injection of mRNA is the most often employed. However, one-cell stage injection is not fully suitable for studying dosing regimens and the duration of protein half-life in differentiated cells. While there are currently no comprehensive studies comparing mRNA stability between the zebrafish and the mammals, the fast division of the embryo during the first 24 h post-transfection dilutes the mRNA rapidly. This has resulted in the visible protein disappearing quickly in the zebrafish embryo as compared with in vitro cell models [13]. If the zebrafish is to be used to test potential delivery vehicles before moving on to the rodent models, the desired target organ for mRNA delivery in older embryos also needs to be evaluated, as homologues of the human/rodent target receptors may or may not be present in the zebrafish embryo. Furthermore, even if a homologue exists, it is possible that the receptor is expressed in other tissues. Notably, one study showed that anionic LNPs could efficiently transfect sinusoidal endothelial cells via the stabilin-1 and stabilin-2 receptor, mimicking delivery to the liver. However, these endothelial cells in the zebrafish are not primarily found in the liver, as is the case in mammals, but reside in the scavenging blood vessels [29,42]. Finally, zebrafish larvae are not suitable to address the full range of immunogenicity of the mRNA-based approach, in particular when using a delivery vehicle. While reports have been made of hypersensitivity reactions to PEG-coated LNPs in humans, the zebrafish embryo does not possess an adaptive immune system until 5–6 weeks post-fertilization [42,43].

## 7. The Translatability of Zebrafish Studies to Human Diseases

As mentioned, no mRNA-based therapies for protein replacement that were tested in zebrafish have found their way into clinics yet. Therefore, there are no extensive studies comparing the kinetics, translation efficiency and immunogenicity between the zebrafish model and the human disease, and we can only compare what is observed in zebrafish and mammal models (see above).

However, the zebrafish has emerged in recent years as a powerful preclinical model to study human disease, with phenotype characteristics and molecular mechanisms being highly conserved in these models. Second, zebrafish show a remarkable degree of similarity of physiological functions. Some physiological processes are even more comparable between humans and zebrafish, as opposed to the comparison between humans and rodents (for example: cardiac electrophysiology). Next to studying human disease, the zebrafish has also gained momentum as a model for drug screening, as many drugs with known effects in humans have been shown to elicit similar effects in the zebrafish embryo [39], such as psychoactive compounds, anti-angiogenic drugs, cardiovascular treatments and anti-cancer drugs [44]. Furthermore, it has been shown that zebrafish responds to small molecule drugs at the physiologically relevant doses, enabling high-throughput and high-content drug screening. Most importantly, zebrafish assays can support drug development by structure–activity profiling and toxicity screening, often in combination with testing in other models [37]. As mentioned, only innate immunity responses can be studied in the zebrafish, meaning that additional studies in older fish or other animal models will be needed to study adaptive immune responses that are translatable to the human setting. While it is unlikely that the zebrafish model can fully replace rodent testing, the use of zebrafish (embryonic) models as an intermediate step between the in vitro and mammal models can greatly reduce the number of animals needed for the development of (mRNA-based) therapies.

## 8. Conclusions

The injection of genetic material into the zebrafish is a prevalent method in preclinical research but has not been extensively applied yet in the context of mRNA-based protein replacement therapies. We illustrate that zebrafish disease models can be used for the early in vivo testing of mRNA-based approaches, by generating reliable data regarding effectiveness in a high-throughput manner after one-cell stage injection. Furthermore, the zebrafish embryos at later life stages (>24 hpf, <120 hpf) can be used for evaluation of delivery vehicles after injection into the circulatory system. Altogether, while the zebrafish larvae model cannot completely replace higher animal models yet, they can serve as the first cost-effective step for testing mRNA-based therapies prior to more expensive and time-consuming studies in mammals.

## Figures and Tables

**Table 1:** The characteristics of DNA- and mRNA-based protein replacement therapies and viral versus non-viral delivery strategies.

DNA-Based Protein Replacement	mRNA-Based Protein Replacement
Not mutation specific	Not mutation specific
Nuclear entry required	Functional in cytoplasm
Transcription and translation	Direct translation
Long-term	Transient
Risk of mutagenesis after genomic integration	No genomic integration
**Viral vectors**	**Non-viral delivery vehicles**
High transduction efficiency	More efficient for non-dividing cells
Limited cell-specific targeting	More options for specific delivery
Size restrictions of transgene	No size restrictions
Highly immunogenic	Biodegradable
Difficult to manufacture	Simple manufacturing (low cost)

Protein replacement can be performed by DNA-based gene therapy or by mRNA-based approaches, each having their own pros and cons. In general, DNA-based approaches utilize viral vector-based gene delivery, while mRNA-based approaches most often employ non-viral vehicles.

**Table 2:** General comparison between in vitro cell models, zebrafish embryos and rodent models, with special attention paid to their potential for the testing of mRNA-based therapeutics.

In Vitro Models	Zebrafish Larval Models	Rodent Models
Low maintenance cost	Low maintenance cost	High maintenance cost
Simplified	Moderate difficulty	High difficulty
Short timeframe	Short timeframe	Long-term experiments
Poor translatability	Moderate translatability	High translatability
High flexibility	Moderate flexibility	Low flexibility
Unrealistic cellular morphology and interactions	Genetic similarity to humans	Genetic similarity to humans
High throughput	High throughput	Lower throughput
Easy genetic modulation	Easy genetic modulation	Complex genetic modulation
Rapid genetic rescue	Rapid genetic rescue	More complex genetic rescue
Naked or packaged mRNA	Naked or packaged mRNA	Preferably packaged mRNA
Direct transfection of all cells	Ubiquitous expression (one-cell stage injection)	Restricted expression (vehicle dependent)
Effectivity of mRNA-based therapy	Effectivity + delivery of mRNA-based therapy	Effectivity + delivery of mRNA-based therapy
Low to moderate ethical considerations	Low ethical considerations (<120 hpf in Europe)	High ethical considerations

Hpf = hours post-fertilization.

**Table 3:** The current applications of mRNA-based therapies to treat genetic diseases in zebrafish.

Disease	Zebrafish Model	mRNA	mRNA Species	mRNA Form	Injection	Effect	Organ of Interest	Ref.
Duchenne muscular dystrophy	*sapje* *; *sapje-like* **	*hmox1* (P)	Zebrafish	Naked	One-cell	Restored skeletal muscle structure	Muscle	[21]
Wolfram Syndrome	*wfs1ab^KO^*	*Ncs1* (P)	Mouse	Naked	One-cell	Decreased hyperlocomotion and restored mitochondrial function	Muscle	[12]
Classic galactosemia	*galt^KO^*	*GALT* (C)	Human	Naked + LNP	One-cell + Intravenous (48 hpf)	Restored enzyme activity and reduced accumulation of metabolites	Liver	[11]
Cystinosis	*ctns^KO^*	*CTNS* (C)	Human	Naked	One-cell	Reduced cystine accumulation and restored pronephros function	Kidney	[13]

For a select few genetic diseases, a zebrafish disease model has been injected with mRNA to restore the phenotype. In the zebrafish, (packaged) mRNA derived from human, zebrafish and mouse sequences can be injected. The one-cell stage is the most widely applied method and leads to ubiquitous expression. However, later-stage injections also show potential but mainly accumulate in the liver. * Point mutation (*sap*^ta222a^), ** splicing mutant (*sap^c/100^*), P = gene playing role in pathogenesis, C = causative gene, LNP = lipid-based nanoparticle, hpf = hours post-fertilization.

## Data Availability

No new research data were generated for this review.
